# Supplementary material for: A retrospective clinical, multi-center cross-sectional study to assess the severity and sequela of Noma/Cancrum oris in Ethiopia
Source: PLoS Negl Trop Dis. 2022 Sep 13;16(9):e0010372. doi: 10.1371/journal.pntd.0010372 (PMC9506604; doi:10.1371/journal.pntd.0010372)

Supporting Information

S1 Appendix: A modified case report form (MCRF) consisting of demographic and clinical information.


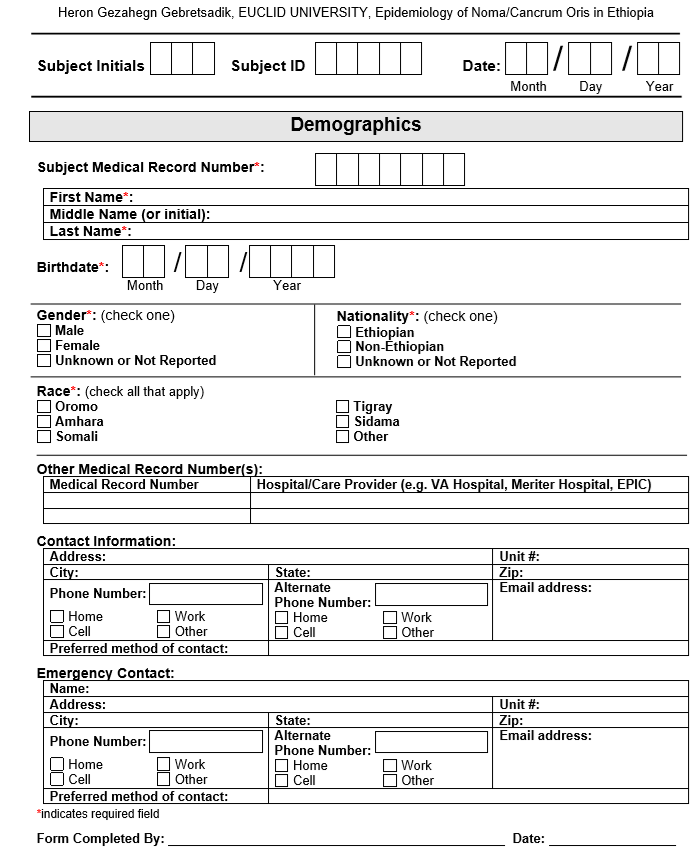


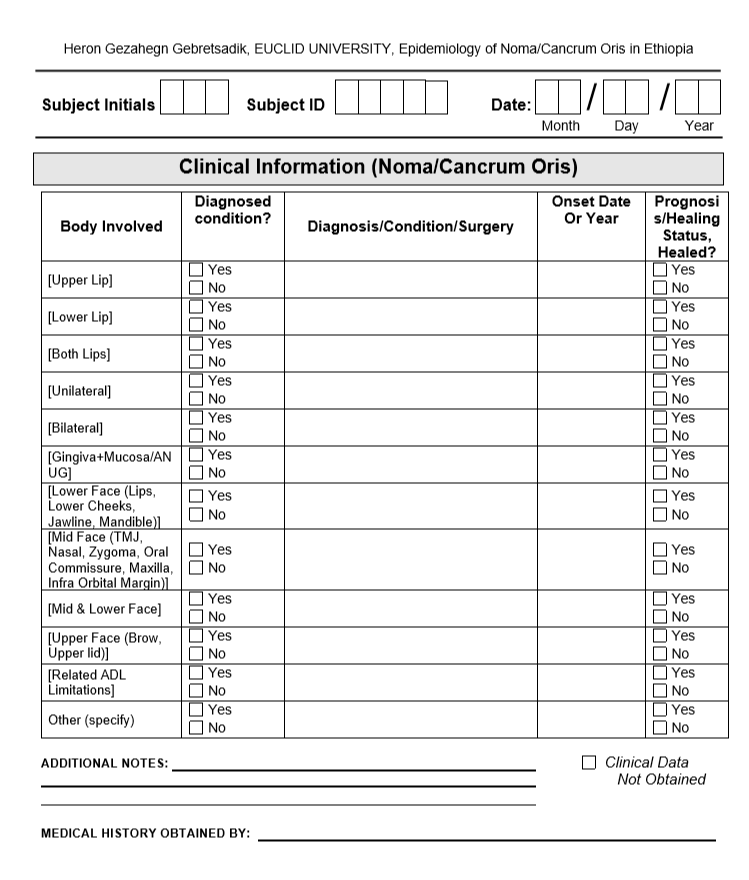

Supplement: S1 Appendix — (DOCX) [file pntd.0010372.s001.docx]
