# Supplementary material for: A retrospective clinical, multi-center cross-sectional study to assess the severity and sequela of Noma/Cancrum oris in Ethiopia
Source: PLoS Negl Trop Dis. 2022 Sep 13;16(9):e0010372. doi: 10.1371/journal.pntd.0010372 (PMC9506604; doi:10.1371/journal.pntd.0010372)
Supplement: Attachment — Submitted filename: Cancrum Oris review.docx [file pntd.0010372.s002.docx]

A REVIEW

A retrospective clinical, multi-center cross-sectional study to assess the severity and sequela of Noma/Cancrum oris in Ethiopia

Overall: The work of the authors is commendable. Despite the debilitating nature of cancrum oris, information about the disease is sparse and hence the study is very relevant to the advancement of knowledge on the subject.

However the authors need to do a substantial revision for the acceptability as a scientific paper.

1. Introduction: This is a section the authors supposed to enlighten the reading audience about the information available on the subject matter and hence citing the former works on the subject. However apart from reference 1-8 which appeared on about one third of the introductory part, the sentences were not referenced.
2. Methodology : Fair but no words on the software employed for analysis
3. RESULTS

Some Observation:

“All the retrospectively 138 studied study participants” a sentence like this affected the flow of the write up. There is a need to appreciate that once the study method has been mentioned, generated data should be used freely example “138 participants were studied”

I am of the opinion that a table summarizing left sided and right sided deformities will be easier to follow.

Also when using a table, its important to mention it at the beginning of the sentence rather than the end. EG Table 1 shows the distribution of facial defects on the right side of the face observed from the study population

Ethics is always a controversial issue. However blocking the face in these subjects has substancially affected the true extent and appreciation and hence limiting how much the public need to be educated. So I am of the opinion that the face should be presented following informed consent. I appreciate the fact that it is a retrospective study but all surgeons are married to our patients and I am sure some of them will still be around.

4. Discussion

This section looks like another literature review. I think we can have a better write up if authous focus more on discussing their result in relation to existing literatures. Some of the references could be taken to the introductory section.

5. Conclusion

What was presented was not a summary. Conclusion should be the opinion arrived from discussion. The write up looks like another view. A revision is needed.

“Noma is a debilitating disease primarily affecting the poor and has been called the “face of poverty.” It occurs among the underprivileged society where patients do not have access to proper medical care. Those who do develop Noma are doomed to suffer the tragic consequences of disfigurement and functional impairment, or death.” These are established facts from past studies, so cant be a conclusion of this study

REfernces: They are in order. Authors should ensure they are in line with the PLOS journal

Best of Luck.
