# Supplementary material for: A retrospective clinical, multi-center cross-sectional study to assess the severity and sequela of Noma/Cancrum oris in Ethiopia
Source: PLoS Negl Trop Dis. 2022 Sep 13;16(9):e0010372. doi: 10.1371/journal.pntd.0010372 (PMC9506604; doi:10.1371/journal.pntd.0010372)
Supplement: Attachment — Submitted filename: REvision Review PNTD 22.docx [file pntd.0010372.s004.docx]

REVISION

A retrospective clinical, multi-center cross-sectional study to assess the severity and sequela of Noma/Cancrum oris in Ethiopia

The effort of the authors is highly commendable. There is substantial improvement of the article.

Recommendation: The paper is recommended for publication with minor revision highlighted below.

1. Table……………………………Line tables are not acceptable in scientific writing. PLs employ no line designs to eliminate the lines
2. Dental involvement has been reported in the majority of medical records reviewed in this study. Out of the total 163 medical records reviewed, 7.4% (n=12) had no dental 266 information………….In scientific reseach , source of information could be medical records also known as case file or a proforma. But once inforemation are retrieved, these sources are dropped and we focus on the subjects. The above statement should be repframed as follows:

Dental involvement has been reported in the majority of the patients in this study. Out of the

total 163 patients reviewed, 7.4% (n=12) had no dental 266 information

3. Discussion: The authors have shown that they understand how to employ result for discussion, however it takes the reader 26 lines before the mention of the study results. Just a brief introduction is needed before bringing your result. Maximum within 5 to 10 lines.
