# Supplementary material for: A retrospective clinical, multi-center cross-sectional study to assess the severity and sequela of Noma/Cancrum oris in Ethiopia
Source: PLoS Negl Trop Dis. 2022 Sep 13;16(9):e0010372. doi: 10.1371/journal.pntd.0010372 (PMC9506604; doi:10.1371/journal.pntd.0010372)
Supplement: Attachment — Submitted filename: Response to reviewers_PLOS_HGG_LC.docx [file pntd.0010372.s005.docx]

**Response to reviewers**

**PLOS Neglected Tropical Diseases**

**Title**: A retrospective clinical, multi-center cross-sectional study to assess the severity and sequela of Noma/Cancrum oris in Ethiopia

**Manuscript Number: PNTD-D-22-00410**

*Dear Joseph M. Vinetz,*

*We are delighted that our manuscript has been reviewed for publication by PLOS Neglected Tropical Diseases. Thank you very much for your constructive feedback and helpful guidance.*

*We have taken the reviewers’ comments very seriously and have invested a great amount of work to adequately address the concerns described in your decision email. We copied the reviewers’ comments as presented in the email. For clarity, we bolded the reviewers’ comments as presented below. We responded to the reviewer comments individually, indicating exactly how we addressed each concern or problem and describing the changes made. Two versions of the revised manuscript: one with highlights denoting where the text has been changed; the other a clean version are uploaded as the manuscript file. Contents removed and added to the highlighted manuscript are colored in red and green, respectively. We hope that you approve the taken steps and look forward to hearing from you soon.*

*The authors.*

*2. METHODOLOGY*

***Comment of Reviewer #1***

***Fair but no words on the software employed for analysis***

*Thank you very much for your valuable and very helpful comment. The analysis was employed using the SPSS software program and is now described in the materials and methods section of the manuscript.*

*3. RESULTS*

***Comments of Reviewer #1***

***“All the retrospectively 138 studied study participants” a sentence like this affected the flow of the write up. There is a need to appreciate that once the study method has been mentioned, generated data should be used freely example “138 participants were studied”***

*Thank you for making us aware of this point. We adapted this specific and other similar sentences in our entire manuscript based on your recommendation.*

***I am of the opinion that a table summarizing left-sided and right-sided deformities will be easier to follow.***

*Thank you very much for your suggestion. We fully agree with your comment to summarize the left-sided and right-sided deformities in a table. Accordingly, we brought a new table (Table 1) to your attention. The table describes better the presentation of the overall facial deformities documented in the manuscript.*

***Also when using a table, its important to mention it at the beginning of the sentence rather than the end. EG Table 1 shows the distribution of facial defects on the right side of the face observed from the study population***

*Thank you for this comment. We applied the recommendation for all 3 tables included in our manuscript.*

***Ethics is always a controversial issue. However blocking the face in these subjects has substancially affected the true extent and appreciation and hence limiting how much the public need to be educated. So I am of the opinion that the face should be presented following informed consent. I appreciate the fact that it is a retrospective study but all surgeons are married to our patients and I am sure some of them will still be around.***

*Thank you for highlighting this important aspect. Accordingly, we unblock the faces of the subjects included in our manuscripts.*

***Comment of Reviewer #2***

***Yes - most images are good quality - 1 noticeably blurry - on the cut-off for what I would consider acceptable for an image***

*Thank you for bringing this important point to our attention. We checked the quality of all pictures using the Preflight Analysis and Conversion Engine (PACE) digital diagnostic tool to meet PLOS requirements. Accordingly, we removed Figures 2 and 5 that failed to meet PLOS requirements following the PACE check. On the other hand, we included two additional new figures (5 and 6) to enhance the visual content of our manuscript. These figures fulfilled the PLOS requirements.*

***Editorial comment***

***Table……………………………Line tables are not acceptable in scientific writing. PLs employ no line designs to eliminate the lines***

***Comment of Reviewer #1***

***Analysis and figures well presented. The tables should be change dfrom Line design to non line design***

*Thank you for your important comments. Based on both recommendations, the lines of all the tables in the manuscript are removed. Accordingly, no line design is employed.*

*Editorial comment*

***Dental involvement has been reported in the majority of medical records reviewed in this  study. Out of the total 163 medical records reviewed, 7.4% (n=12) had no dental 266 information………….In  scientific reseach , source of information could be medical records also known as case file or  a proforma. But once inforemation are retrieved, these sources are dropped and we focus on the subjects.  The above statement should be repframed as follows:
 Dental involvement has been reported in the majority of the patients in this study. Out of the  total 163 patients reviewed, 7.4% (n=12) had no dental 266 information***

*Thank you very much for this useful suggestion. The statement is reframed in the manuscript based on the comment.*

*5. DISCUSSION*

***Editorial comment***

***Discussion: The authors have shown that they understand how to employ result for discussion, however it takes the reader 26 lines before the mention of the study results. Just a brief introduction is needed before bringing your result. Maximum within 5 to 10 lines****.*

*Once again, thank you very much for this constructive suggestion. The recommended change has been made in the manuscript based on this helpful comment.*

*6. CONCLUSION*

***Comments of Reviewer #1***

***What was presented was not a summary. Conclusion should be the opinion arrived from discussion. The write up looks like another view. A revision is needed.***

***“Noma is a debilitating disease primarily affecting the poor and has been called the “face of poverty.” It occurs among the underprivileged society where patients do not have access to proper medical care. Those who do develop Noma are doomed to suffer the tragic consequences of disfigurement and functional impairment, or death.” These are established facts from past studies, so cant be a conclusion of this study***

*Thank you for this valuable and helpful comment. We have gone through the conclusion section of our manuscript and made the needed changes.*

***Comments of Reviewer #2***

***yes - writing needs to be improved - I have made the majority of required grammatical changes. This manuscript will require another proofread to make sure no errors remain.***

*We agree with your comments. We carefully revised the writing to improve the manuscript based on your valuable comments. Accordingly, we have gone through the entire manuscript carefully and adjusted every issue that needs to be addressed.*

*EDITORIAL AND DATA PRESENTATION MODIFICATIONS?*

***Comment of Reviewer #1***

***Substantial revision important***

*Thank you for your comment. We revised the manuscript substantially based on all recommendations forwarded to us.*

*SUMMARY AND GENERAL COMMENTS*

***Comment of Reviewer #1***

***Overall: The work of the authors is commendable. Despite the debilitating nature of cancrum oris, information about the disease is sparse and hence the study is very relevant to the advancement of knowledge on the subject. However substantial revision is needed to be acceptable as a scientific publication.***

*We would like to thank you for your very positive feedback. We revised our manuscript carefully to maximize its quality as a scientific publication.*

***Comments of Reviewer #2***

***Thank you for your submission.***

***Detailed review. Would make changes to the manuscript as follows:***

*Thank you very much for your detailed revision of our manuscripts. We also appreciate your insightful comments on all aspects of the manuscript including the abstract. We made big efforts to address all the issues* *point-by-point and improve our manuscript.*

*Once again,* *thank you very much for your most constructive and helpful comments. We appreciate the opportunity to revise our manuscript.*

*The authors.*
